# The relationship between skeletal muscle mass and cardiac structure in elderly male inpatients

**DOI:** 10.3389/fcvm.2026.1923652

**Published:** 2026-08-26

**Authors:** Lingyan Chen, Le Song, Xi Li, Yu Hu, Man Luo

**Affiliations:** 1 Geriatric Department, Geriatric Medical Centre, Shanghai, China; 2 Geriatric Department, Zhognshan Hospital, Fudan University, Shanghai, China; 3 International Medical Center, Zhognshan Hospital, Fudan University, Shanghai, China

**Keywords:** echocardiography, handgrip strength, left ventricular structure, sarcopenia, skeletal muscle mass index

## Abstract

**Introduction:**

To investigate the associations of skeletal muscle mass index (SMI) and handgrip strength with cardiac structure and function in elderly male inpatients without severe acute cardiovascular conditions.

**Methods:**

This cross-sectional study included 143 male inpatients aged ≥60 years. SMI was assessed using dual-energy x-ray absorptiometry, handgrip strength was measured by electronic dynamometry, and cardiac structure and function were evaluated by two-dimensional echocardiography. Multivariable linear regression and restricted cubic spline models were performed to assess the associations between skeletal muscle characteristics and echocardiographic parameters.

**Results:**

Compared with the normal-SMI group, participants with low SMI had significantly smaller ARD, LVEDD, and LVESD (all *P* < 0.05). In fully adjusted models, SMI remained independently associated with ARD (*β* = 0.131, *P* = 0.048), LVEDD (*β* = 0.135, *P* = 0.032), LVESD (*β* = 0.152, *P* = 0.013), and LVEF (*β* = 0.130, *P* = 0.035). Handgrip strength was independently associated only with ARD (*β* = 0.283, *P* = 0.009). Restricted cubic spline analyses demonstrated linear associations between SMI and left ventricular parameters (all *P* for non-linearity > 0.05). Subgroup analyses showed consistent associations across predefined strata without significant interactions.

**Discussion:**

In elderly male inpatients, SMI was independently and linearly associated with left ventricular structure and systolic function, whereas handgrip strength showed fewer significant associations with cardiac parameters. These observational findings suggest that SMI may represent a potential marker of age-related left ventricular structural changes; however, longitudinal studies are required to determine the temporal relationship and clinical implications.

## 1 Introduction

The concurrent rise in global life expectancy and the increasing burden of age-related chronic diseases have made sarcopenia and cardiovascular disease (CVD) two of the most pressing public health challenges of the twenty-first century. Sarcopenia, defined as the progressive and generalized loss of skeletal muscle mass, strength, and function, affects approximately 10%–27% of older adults worldwide, with a prevalence of 10%–22% in the Chinese population (1–3). Although its onset is often insidious, sarcopenia profoundly undermines physical capability, functional independence, and quality of life, and is consistently associated with a heightened risk of falls, fractures, delirium, rehospitalization, prolonged hospital stays, postoperative complications, and all-cause mortality in older patients (4). Given the accelerating pace of population aging, identifying effective strategies to mitigate the adverse health consequences of sarcopenia has become an urgent research priority.

Aging is characterized by a complex interplay of systemic alterations, including hormonal dysregulation, mitochondrial dysfunction, insulin resistance, oxidative stress, and chronic low-grade inflammation (5). These same mechanisms are also central to the pathogenesis of CVD, and advancing age serves as the dominant risk factor for both sarcopenia and CVD (6). This shared biological substrate has prompted growing interest in the so-called “muscle–heart axis”—a bidirectional crosstalk in which skeletal muscle depletion and cardiac disease may mutually influence each other's progression (7). Clinically, generalized muscle wasting is frequently observed in patients with heart failure, and conversely, reduced handgrip strength has been associated with an increased risk of coronary heart disease (CHD) in the Framingham Heart Study (8, 9). Moreover, skeletal muscle mass has been shown to correlate most strongly with peak oxygen consumption, and low muscle mass independently predicts higher all-cause mortality in CHD patients (10). These observations strongly suggest that skeletal muscle status is not merely a bystander in cardiovascular health but may actively participate in the pathophysiological processes that determine cardiac outcomes.

At the same time, the aging heart undergoes profound structural and functional remodeling. The most characteristic change is progressive left ventricular thickening, which reflects both a physiological adaptation to age-related hemodynamic shifts and a maladaptive process that predisposes to CVD (7, 11). At the cellular level, autopsy studies have revealed that in elderly patients without clinically apparent CVD, cardiomyocyte hypertrophy coexists with a significant reduction in the total number of myocytes, accompanied by increased interstitial fibrosis (12). These senescent alterations—collectively termed cardiac involution—result in reduced chamber compliance and, eventually, impaired systolic and diastolic performance. Several recent investigations have begun to explore the link between sarcopenia and cardiac remodeling. Previous studies have suggested that sarcopenia is associated with adverse cardiac remodeling and impaired cardiac function in older adults. Lower skeletal muscle mass and strength have been linked to alterations in cardiac structure, supporting the existence of a muscle–heart interaction during aging (13). Similarly, Beyer et al. found that handgrip strength was positively correlated with left ventricular diastolic volume in a community-based UK cohort (14). While these findings support an association between skeletal muscle status and cardiac structure, the evidence remains fragmented and, in some respects, inconsistent.

Crucially, most previous studies have focused either on muscle mass or on muscle strength in isolation, leaving it unclear which of these two dimensions—structural or functional—exhibits the stronger and more independent relationship with cardiac remodeling. Moreover, the available data have been derived predominantly from community-dwelling populations or patients with established CVD, with relatively little attention paid to hospitalized elderly individuals who do not have severe acute cardiac conditions. In this latter group, the dominant cardiac phenotype is more likely to reflect age-related involution rather than ischemia-driven remodeling, and the muscle-heart association may therefore manifest differently. Elucidating this relationship is clinically important because, if muscle mass proves more closely and independently associated with cardiac structure than muscle strength, it would suggest that objective body composition assessment should take precedence over functional testing alone in cardiovascular risk stratification. In addition, exploring whether skeletal muscle preservation correlates with myocardial structural characteristics may generate hypotheses for future longitudinal investigations.

Against this background, the present study was designed to systematically evaluate, in a cohort of elderly male inpatients without severe acute cardiovascular conditions, (1) the associations of SMI and handgrip strength with detailed echocardiographic parameters of cardiac structure and function, and (2) the relative independence of these two muscle-related measures in predicting left ventricular remodeling. We hypothesized that SMI would demonstrate a more consistent and robust association with cardiac chamber dimensions and systolic function than handgrip strength, reflecting a closer biological coupling between skeletal and myocardial muscle mass in the context of aging.

## 2 Participants and methods

### 2.1 Study design and participants

This was a cross-sectional, single-center study conducted at the Geriatric Department of Zhongshan Hospital, Fudan University, between October 2018 and March 2021. A total of 188 male inpatients aged 60 years or older were initially screened. Participants were included if they met all of the following criteria: (1) age ≥60 years; (2) presence of at least one chronic condition requiring long-term medical management; (3) stable vital signs at the time of enrollment; (4) complete clinical data documented in the hospital information system; (5) sufficient cognitive and communicative ability to understand and complete the survey instruments; and (6) provision of written informed consent.

Exclusion criteria were: (1) critical illness or terminal status, including multiple organ failure or end-of-life care; (2) occurrence of an acute clinical event within the preceding week, such as acute myocardial infarction, cerebral hemorrhage, massive gastrointestinal bleeding, or severe trauma; (3) severe cognitive impairment precluding cooperation; (4) missing >20% of clinical, laboratory, or questionnaire data; and (5) refusal to participate.

After applying these criteria, 6 patients were excluded because of missing dual-energy x-ray absorptiometry (DXA) data, 23 were excluded due to the presence of malignancy, mobility disorders, severe hepatic or renal dysfunction, severe heart failure, or acute conditions (including infection, acute cerebrovascular stroke, or recent surgical stress), and 16 were excluded owing to incomplete echocardiographic records. The final analytical cohort comprised 143 participants, among whom handgrip strength measurements were available for 127 patients (16 patients were unable to complete handgrip assessment due to severe hand osteoarthritis, post-stroke hemiparesis, or severe cognitive/cooperative limitation).

The study was conducted in accordance with the Declaration of Helsinki (1964) and its later amendments and was approved by the Research Ethics Committee of Zhongshan Hospital, Fudan University (approval No. B2017-079R). Written informed consent was obtained from all participants prior to enrollment.

### 2.2 Data collection and clinical measurements

Demographic characteristics, lifestyle factors, medical history, and current medication use were obtained through standardized interviews and medical record review. Body weight and height were measured with participants wearing light clothing and no shoes, and body mass index (BMI) was calculated as weight divided by height squared (kg/m^2^). Resting blood pressure, including systolic blood pressure (SBP) and diastolic blood pressure (DBP), was measured three times at 5 min intervals using an automated electronic sphygmomanometer (Omron HEM-752 FUZZY, Omron Co., Dalian, China) after at least 10 min of rest in the seated position; the average of the three readings was used for analysis.

Fasting venous blood samples were drawn after an overnight fast of at least 10 h. Biochemical parameters, including fasting blood glucose (FBG), total cholesterol (TC), triglycerides (TG), and high-density lipoprotein cholesterol (HDL-C), were measured using an automated biochemical analyzer (Cobas c702, Roche Diagnostics, Germany). Low-density lipoprotein cholesterol (LDL-C) was calculated using the Friedewald equation.

### 2.3 Body composition assessment

Whole-body and regional body composition, including lean mass, fat mass, and bone mineral density, were measured using a DXA scanner (Discovery, S/N 88576, Hologic, Inc., Bedford, MA, USA). All DXA scans were performed and analyzed by a single trained technician following a standardized protocol, using the manufacturer's manual analysis software. Appendicular skeletal muscle mass (ASM) was defined as the sum of lean soft tissue mass in both upper and lower limbs. The SMI was calculated as ASM divided by height squared (kg/m²).

Handgrip strength was measured using an electronic hand dynamometer (CAMRY EH101, Xiangshan, China). Participants were instructed to stand upright with their arms hanging naturally at their sides and to perform maximum voluntary contraction with their dominant hand. The measurement was repeated twice, with a 1 min rest interval between attempts. The average value of the two readings, recorded to the nearest 0.1 kg, was used for analysis.

### 2.4 Echocardiographic examination

Transthoracic echocardiography was performed using a Philips EPIC 7C ultrasound system (Philips Ultrasound, Bothell, WA, USA) equipped with a 2–4 MHz phased-array transducer. Participants were placed in the left lateral decubitus position (approximately 30° tilt). All echocardiographic measurements were obtained according to the American Society of Echocardiography guidelines, using two-dimensional and targeted M-mode imaging with Doppler color flow mapping. Standard parasternal long-axis and apical four-chamber and two-chamber views were acquired.

Measured structural parameters included: aortic root diameter (ARD), left atrial diameter (LAD), left ventricular end-diastolic diameter (LVEDD), left ventricular end-systolic diameter (LVESD), interventricular septal thickness (IVST), left ventricular posterior wall thickness (LVPWT), and pulmonary artery systolic pressure (PASP). Left ventricular ejection fraction (LVEF) was calculated using the biplane modified Simpson's method from the apical four- and two-chamber views. All measurements were performed by experienced echocardiographers who were blinded to the participants' clinical and body composition data.

### 2.5 Definitions of clinical conditions

Low SMI was defined according to the criteria of the Asian Working Group for Sarcopenia (AWGS) 2019 consensus, with a cutoff value of <7.0 kg/m^2^ for men (15).

Osteoporosis was defined as a bone mineral density T-score ≤−2.5 standard deviations below the young adult mean, according to World Health Organization criteria (16). Obesity was defined as BMI ≥28 kg/m^2^, the recommended cutoff for Chinese adults (17).

Diabetes mellitus (DM) was defined according to the 1999 World Health Organization diagnostic criteria (18), or based on a documented history of diabetes or current use of glucose-lowering medications. Hypertension was defined as SBP ≥140 mmHg and/or DBP ≥90 mmHg, or self-reported use of antihypertensive agents (19). Coronary heart disease (CHD) was defined based on a documented history of myocardial infarction, acute coronary syndrome, or the presence of ≥50% stenosis in at least one major coronary artery confirmed by coronary computed tomography angiography or invasive coronary angiography.

### 2.6 Statistical analysis

Continuous variables are presented as mean ± standard deviation (SD) for normally distributed data, or as median with interquartile range (P25, P75) for skewed variables. Categorical variables are expressed as numbers and percentages. Comparisons between groups (control vs. low SMI) were performed using one-way analysis of variance (ANOVA) or the Kruskal–Wallis test for continuous variables, and the chi-square test for categorical variables, as appropriate.

Pearson correlation analysis was used to examine the unadjusted linear associations between SMI, handgrip strength, and echocardiographic parameters.

To evaluate the independent associations of SMI and handgrip strength with cardiac structural and functional parameters, we performed multivariable linear regression analyses with three sequential adjustment models:

- Model 1: adjusted for age and BMI;
- Model 2: additionally adjusted for a history of CHD;
- Model 3: further adjusted for DM and hypertension (full adjustment model).

All covariates included in the regression models were selected *a priori* based on biological plausibility and previous literature. Multicollinearity was rigorously evaluated using the variance inflation factor (VIF); all VIF values across multivariable models were <2.5 (ranging from 1.08 to 2.14), confirming no significant collinearity. To evaluate potential model overfitting given our modest sample size (*n* = 143 overall; *n* = 127 for handgrip models), internal validation was performed using nonparametric bootstrap resampling with 1,000 iterations to derive robust 95% confidence intervals (CIs) for regression coefficients (*β*).

For missing data handling, Little's MCAR test confirmed that handgrip strength measurements were missing completely at random (*P* = 0.428). Primary analyses utilized complete-case analysis (*n* = 127 for handgrip strength). Furthermore, sensitivity analyses using multiple imputation by chained equations (MICE, 10 imputed datasets) were performed to assess the potential impact of missing values on primary estimates.

To explore the shape of the dose-response relationship between SMI and echocardiographic parameters, we fitted restricted cubic spline (RCS) regression models with three knots placed at the 5th, 50th, and 95th percentiles of the SMI distribution, using Model 3 covariate adjustment. The nonlinearity of the association was tested by an *F*-test for the null hypothesis that the coefficients of the spline terms are jointly equal to zero; a P-value for non-linearity >0.05 was taken to indicate a linear relationship.

All statistical tests were two-sided, and a *P*-value <0.05 was considered statistically significant. Analyses were performed using SPSS version 26.0 (SPSS, Inc., Chicago, IL, USA).

## 3 Results

### 3.1 Characteristics of the subjects

Finally, 143 participants were included in the analysis, and handgrip data were available for 127 patients. Their general characteristics are shown in Table 1.

**Table 1:** Baseline characteristics of study participants stratified by skeletal muscle index (SMI).

Variable	Total group (*n* = 143)	Control group (*n* = 101)	Low SMI group (*n* = 42)	*P* value
Age (years)	82.45 ± 8.54	81.27 ± 8.19	86.29 ± 6.12	0.002
BMI (kg/m^2^)	24.31 ± 3.25	25.19 ± 3.07	21.39 ± 1.80	<0.001
SBP (mmHg)	134.92 ± 14.44	134.98 ± 14.52	133.84 ± 14.38	0.701
DBP (mmHg)	73.42 ± 9.67	73.47 ± 10.32	73.26 ± 7.23	0.913
FBG (mmol/L)	6.06 ± 1.89	6.19 ± 1.97	5.62 ± 1.52	0.141
TC (mmol/L)	3.77 ± 0.78	3.74 ± 0.76	3.85 ± 0.86	0.486
TG (mmol/L)	1.26 ± 0.70	1.28 ± 0.73	1.18 ± 0.61	0.471
LDL-C (mmol/L)	1.96 ± 0.72	1.97 ± 0.68	1.94 ± 0.84	0.822
HDL-C (mmol/L)	1.23 ± 0.35	1.19 ± 0.33	1.37 ± 0.39	0.011
ARD (mm)	35.38 ± 2.94	35.66 ± 2.78	34.45 ± 3.30	0.045
LAD (mm)	41.54 ± 5.39	41.97 ± 5.45	40.09 ± 4.99	0.079
LVEDD (mm)	46.35 ± 4.62	46.92 ± 4.51	44.45 ± 4.54	0.009
LVESD (mm)	30.06 ± 4.44	30.66 ± 4.51	28.06 ± 3.60	0.004
IVST (mm)	10.82 ± 3.35	10.90 ± 3.69	10.54 ± 1.80	0.607
LVPWT (mm)	9.75 ± 0.98	9.76 ± 0.98	9.71 ± 0.97	0.813
PASP (mmHg)	36.28 ± 8.10	35.10 ± 8.03	40.19 ± 7.15	0.002
LVEF (%)	63.85 ± 6.12	64.38 ± 5.94	62.57 ± 6.41	0.114
SMI (kg/m2)	7.65 ± 0.97	8.02 ± 0.73	6.36 ± 0.56	<0.001
TLM (kg)	54.54 ± 7.51	57.03 ± 6.71	46.66 ± 5.49	<0.001
ALM (kg)	21.89 ± 3.25	23.11 ± 2.80	18.13 ± 2.45	<0.001
FM (%)	21.38 ± 4.86	21.30 ± 4.61	21.97 ± 5.82	0.508
VFM (kg)	1.10 ± 0.12	1.12 ± 0.11	1.03 ± 0.09	<0.001
Handgrip strength (kg), *n* = 127	26.43 ± 6.46	27.56 ± 6.27 (*n* = 97)	22.52 ± 5.61 (*n* = 30)	<0.001
Obesity (n, %)	39 (27.3%)	27 (26.7%)	12 (28.6%)	0.317
Hypertension (n, %)	117 (81.8%)	81 (80.2%)	36 (85.7%)	0.278
DM (n, %)	58 (40.6%)	38 (37.6%)	20 (47.6%)	0.047
Osteoporosis (n, %)	69 (48.3%)	45 (44.6%)	24 (57.1%)	0.045
CHD (n, %)	46 (32.2%)	33 (32.7%)	13 (31.0%)	0.512

Data are expressed as mean ± standard deviation for continuous variables or *n* (%) for categorical variables. Percentages have been systematically recalculated and rounded to one decimal place. LVEF values have been verified against the raw database and corrected.

BMI, body mass index; SBP, systolic blood pressure; DBP, diastolic blood pressure; FBG, fasting blood glucose; TC, total cholesterol; TG, triglycerides; HDL-C, high-density lipoprotein cholesterol; LDL-C, low-density lipoprotein cholesterol; ARD, aortic root diameter; LAD, left atrial diameter; LVEDD, left ventricular end-diastolic diameter; LVESD, left ventricular end-systolic diameter; IVST, interventricular septal thickness; LVPWT, left ventricular posterior wall thickness; PASP, pulmonary artery systolic pressure; LVEF, left ventricular ejection fraction; SMI, skeletal muscle index; TLM, total lean mass; ALM, appendicular lean mass; FM, fat mass; VFM, visceral fat mass; DM, diabetes mellitus; CHD, coronary heart disease.

**Figure 1:**
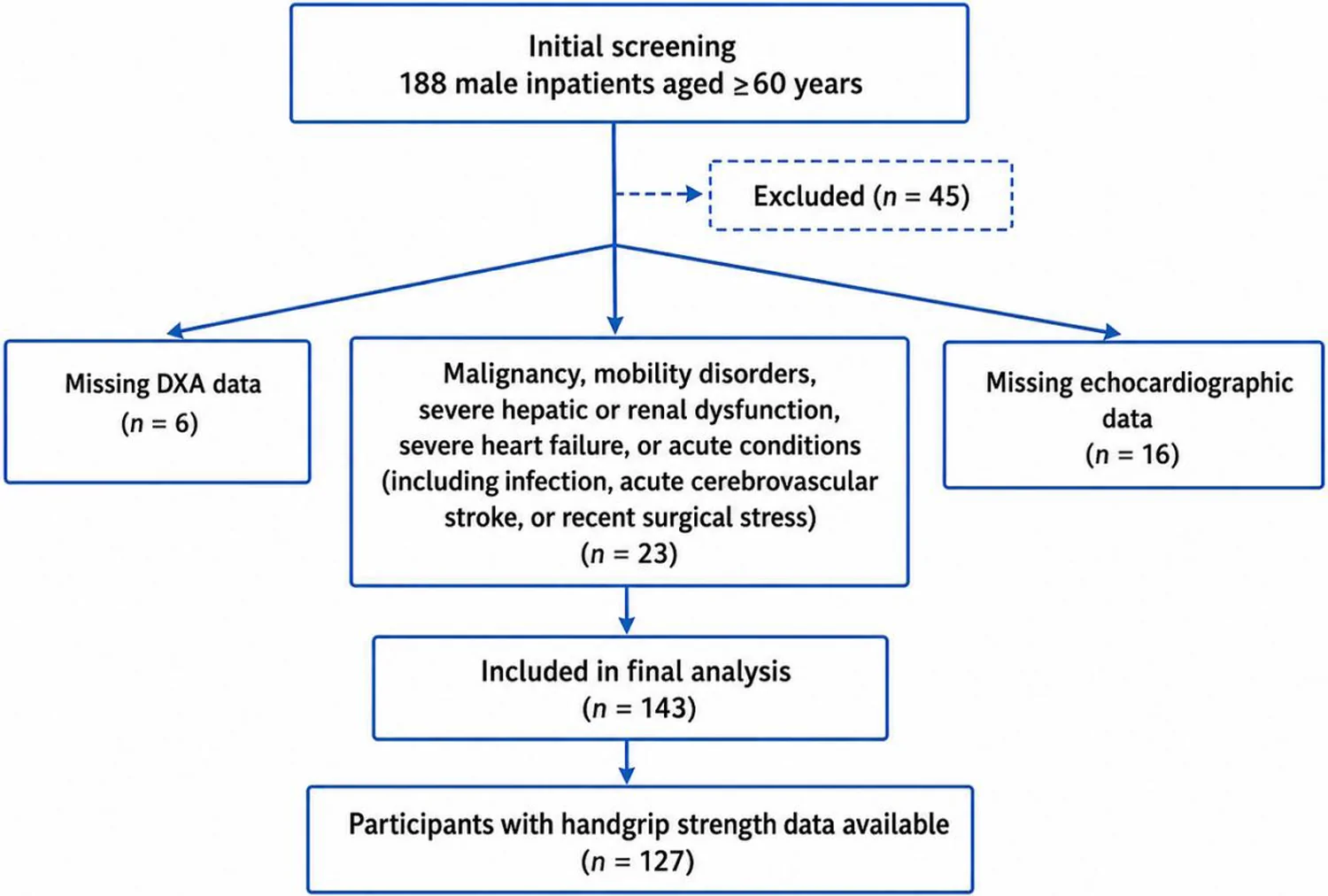
Flowchart of study population selection. Initial screening of 188 male inpatients aged ≥60 years, exclusion criteria, and the final analytical cohort (*n* = 143) with available DXA and echocardiographic data; handgrip strength data were available for 127 participants.

A flowchart illustrating the study selection process is shown in Figure 1. The initial screening included 188 male inpatients. After exclusion of 6 patients due to missing DXA data, 23 due to severe comorbid conditions (malignancy, severe heart failure, or acute critical illness), and 16 due to missing echocardiographic data, a final cohort of 143 participants was included in the analysis. Handgrip strength measurements were available for 127 participants.

### 3.2 Association of SMI with echocardiographic parameters

Compared to the control group, LVEDD, LVESD, and ARD were smaller in the low SMI group, while PASP was higher (*P* < 0.05). As shown in Table 2, Pearson analysis revealed that SMI was positively associated with BMI, TLM, ALM, VFM, LVEDD, LVESD, and LAD, but negatively associated with age and PASP (*P* < 0.05). In addition, handgrip strength was positively associated with BMI, TLM, ALM, VFM, ARD, and LVESD, and negatively associated with age (*P* < 0.05, Table 3).

**Table 2:** Pearson correlation analysis between skeletal muscle index (SMI), handgrip strength, and clinical parameters.

Correlation between SMI and other parameters		
Variable	r value	*P* value
Age (years)	−0.369	<0.001
BMI (kg/m^2^)	0.717	<0.001
TLM (kg)	0.81	<0.001
ALM (kg)	0.894	<0.001
FM (%)	0.021	0.811
VFM (kg)	0.387	<0.001
ARD (mm)	0.153	0.087
LAD (mm)	0.196	0.023
LVEDD (mm)	0.336	<0.001
LVESD (mm)	0.352	<0.001
IVST (mm)	0.089	0.307
LVPWT (mm)	0.085	0.326
PASP (mmHg)	−0.246	<0.001
LVEF (%)	0.055	0.53
Handgrip strength (kg)	0.431	<0.001
Correlation between handgrip strength and other parameters (*N* = 127)		
Variable	*r* value	*P* value
Age (years)	−0.518	<0.001
BMI (kg/m^2^)	0.226	0.006
TLM (kg)	0.388	<0.001
ALM (kg)	0.463	<0.001
SMI	0.431	<0.001
FM (%)	−0.112	0.11
VFM (kg)	0.281	0.001
ARD (mm)	0.167	0.032
LAD (mm)	0.098	0.139
LVEDD (mm)	0.109	0.114
LVESD (mm)	0.184	0.02
IVST (mm)	0.011	0.453
LVPWT (mm)	0.039	0.333
PASP (mmHg)	−0.11	0.112
LVEF (%)	−0.113	0.059

Data are presented as Pearson correlation coefficients (r) and corresponding *P*-values.

SMI, skeletal muscle mass index; BMI, body mass index; TLM, total lean mass; ALM, appendicular lean mass; FM, fat mass percentage; VFM, visceral fat mass; ARD, aortic root diameter; LAD, left atrial diameter; LVEDD, left ventricular end-diastolic diameter; LVESD, left ventricular end-systolic diameter; IVST, interventricular septal thickness; LVPWT, left ventricular posterior wall thickness; PASP, pulmonary artery systolic pressure; LVEF, left ventricular ejection fraction.

**Table 3:** Multivariable linear regression analysis of the associations of skeletal muscle index and handgrip strength with cardiac structure and function parameters.

Variable	Unadjusted *β* (95% CI), *P* value	Model 1 *β* (95% CI), *P* value	Model 2 *β* (95% CI), *P* value	Model 3 *β* (95% CI), *P* value
Association between skeletal muscle index (SMI) and cardiac structure parameters				
ARD	0.175 (0.017–0.333), 0.030	0.113 (0.001–0.225), 0.049	0.108 (−0.005–0.221), 0.062	0.131 (0.001–0.261), 0.048
LAD	0.196 (0.028–0.364), 0.023	0.019 (−0.104–0.142), 0.763	0.004 (−0.116–0.124), 0.946	0.004 (−0.118–0.126), 0.950
LVEDD	0.336 (0.174–0.498), <0.001	0.136 (0.019–0.253), 0.023	0.135 (0.011–0.259), 0.031	0.135 (0.011–0.259), 0.032
LVESD	0.352 (0.192–0.512), <0.001	0.151 (0.035–0.267), 0.011	0.146 (0.029–0.263), 0.015	0.152 (0.034–0.270), 0.013
IVST	0.089 (−0.082–0.260), 0.307	−0.131 (−0.386–0.124), 0.315	−0.143 (−0.399–0.113), 0.275	−0.138 (−0.393–0.117), 0.291
LVPWT	0.085 (−0.083–0.253), 0.326	−0.098 (−0.354–0.158), 0.451	−0.117 (−0.374–0.140), 0.372	−0.115 (−0.370–0.140), 0.373
PASP	−0.246 (−0.412–−0.080), 0.004	0.005 (−0.246–0.256), 0.969	−0.004 (−0.255–0.247), 0.974	−0.004 (−0.256–0.248), 0.973
LVEF	0.055 (−0.116–0.226), 0.530	0.134 (0.019–0.249), 0.022	0.126 (0.008–0.244), 0.038	0.130 (0.010–0.250), 0.035
Association between handgrip strength and cardiac structure parameters (*N* = 127)				
ARD	0.167 (−0.010–0.344), 0.064	0.259 (0.052–0.466), 0.014	0.290 (0.084–0.496), 0.007	0.283 (0.073–0.493), 0.009
LAD	0.098 (−0.080–0.276), 0.279	0.133 (−0.062–0.328), 0.181	0.172 (−0.020–0.364), 0.081	0.169 (−0.026–0.364), 0.096
LVEDD	0.109 (−0.068–0.286), 0.227	0.063 (−0.137–0.263), 0.536	0.100 (−0.101–0.301), 0.328	0.084 (−0.115–0.283), 0.408
LVESD	0.184 (0.009–0.359), 0.041	0.138 (−0.060–0.336), 0.171	0.164 (−0.035–0.363), 0.109	0.154 (−0.045–0.353), 0.132
IVST	0.011 (−0.171–0.193), 0.906	−0.040 (−0.238–0.158), 0.692	−0.019 (−0.217–0.179), 0.855	−0.030 (−0.228–0.168), 0.770
LVPWT	0.039 (−0.140–0.218), 0.667	0.001 (−0.198–0.200), 0.991	0.021 (−0.178–0.220), 0.845	−0.003 (−0.202–0.196), 0.975
PASP	−0.110 (−0.287–0.067), 0.224	0.089 (−0.108–0.286), 0.374	0.094 (−0.104–0.292), 0.356	0.089 (−0.111–0.289), 0.385
LVEF	−0.113 (−0.293–0.067), 0.017	−0.149 (−0.353–0.055), 0.154	−0.106 (−0.311–0.099), 0.308	−0.104 (−0.310–0.102), 0.324

Model 1 was adjusted for age and BMI. Model 2 was additionally adjusted for coronary heart disease (CHD). Model 3 was further adjusted for CHD, diabetes mellitus (DM), and hypertension. Data are presented as regression coefficients (β) with 95% confidence intervals (CIs).

**Figure 2:**
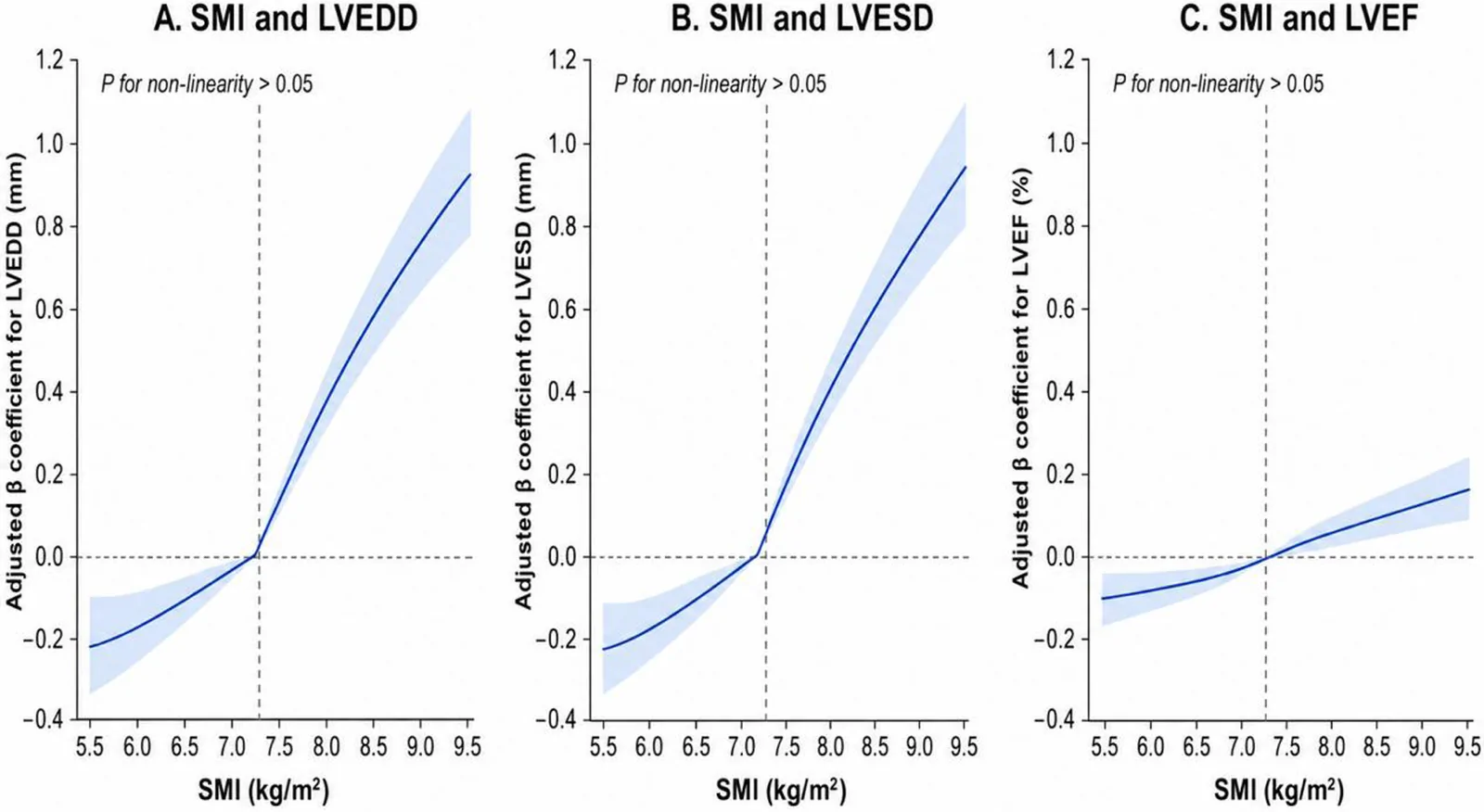
Restricted cubic spline analyses of the associations between skeletal muscle index (SMI) and cardiac structure and function parameters. Restricted cubic spline models were adjusted for age, body mass index (kg/m²), coronary heart disease, diabetes mellitus, and hypertension. Solid lines represent adjusted *β* coefficients, and shaded areas indicate 95% confidence intervals. The vertical dashed line represents the reference value of SMI.

Multivariate linear regression analysis demonstrated that SMI was significantly positively associated with ARD, LVEDD, LVESD, and LVEF, and negatively associated with PASP. After adjustment for age, BMI, coronary heart disease, diabetes mellitus, and hypertension (Model 3), the associations of SMI with ARD (*β* = 0.131, *P* = 0.048), LVEDD (*β* = 0.135, *P* = 0.032), LVESD (*β* = 0.152, *P* = 0.013), and LVEF (*β* = 0.130, *P* = 0.035) remained significant. Handgrip strength remained positively associated with ARD after full adjustment (*β* = 0.283, *P* = 0.009). Bootstrap resampling (1,000 iterations) yielded consistent regression coefficients and narrow 95% CIs across all fully adjusted models, confirming model stability. Sensitivity analyses applying multiple imputation for missing handgrip strength values produced results consistent with the primary complete-case analysis (Figure 2).

We further explored potential nonlinear associations between SMI and cardiac structural parameters using RCS regression models with three knots placed at the 5th, 50th, and 95th percentiles of SMI, after adjustment for the same covariates (Model 3). The RCS analysis demonstrated an approximately linear association between SMI and LVEDD, LVESD, and LVEF (*P* for non-linearity >0.05), indicating that higher SMI was consistently associated with larger ventricular dimensions and better cardiac function across the observed range of muscle mass.

No evidence of a nonlinear, threshold, or U-shaped association was observed. The dose–response curves further confirmed a steady incremental relationship between SMI and cardiac structural parameters, supporting the robustness of the linear regression results.

### 3.3 Subgroup analysis and interaction effects

To evaluate the robustness of the association between SMI and cardiac structural parameters, subgroup analyses and interaction tests were performed.

Subgroup analyses were stratified by age (≤80 years vs. >80 years), hypertension status (yes vs. no), diabetes mellitus (yes vs. no), and coronary heart disease (yes vs. no). The results demonstrated that the positive associations between SMI and left ventricular structural parameters (LVEDD and LVESD), as well as LVEF, were consistent across all predefined subgroups.

**Figure 3:**
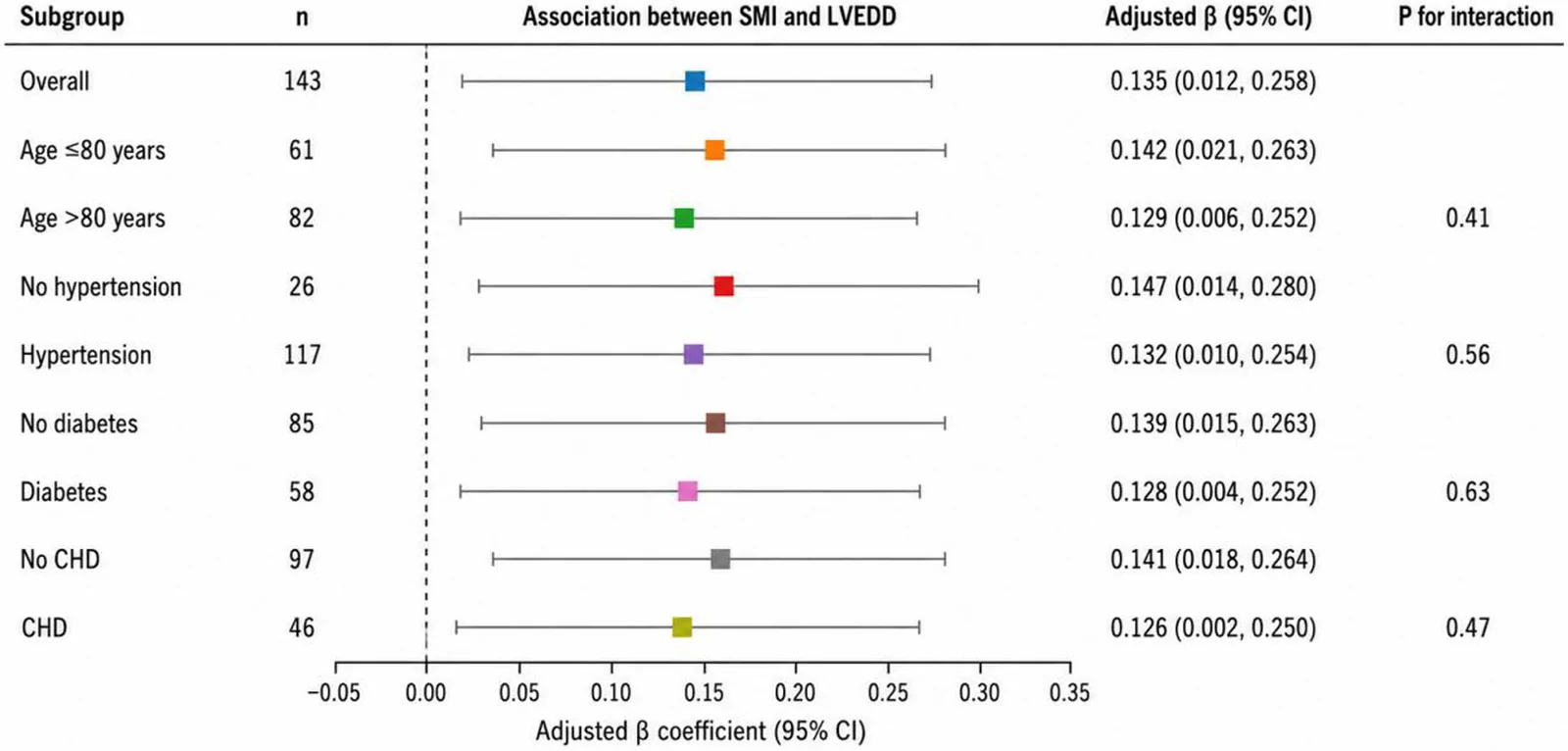
Subgroup forest plot of the association between skeletal muscle index (SMI) and left ventricular end-diastolic diameter (LVEDD). Subgroup analyses were performed according to age, hypertension, diabetes mellitus, and coronary heart disease status. Multivariable linear regression models were adjusted for age, body mass index, coronary heart disease, diabetes mellitus, and hypertension, except for the stratification variable in each subgroup model. Data are presented as adjusted *β* coefficients with 95% confidence intervals (CIs); *P* values for interaction indicate potential effect modification across subgroups. Subgroup sample sizes were as follows: age ≤80 years, *n* = 61; age >80 years, *n* = 82; hypertension, *n* = 117; no hypertension, *n* = 26; diabetes mellitus, *n* = 58; no diabetes mellitus, *n* = 85; coronary heart disease, *n* = 46; no coronary heart disease, *n* = 97.

No statistically significant interaction effects were observed between SMI and age (*P* for interaction = 0.41), hypertension (*P* for interaction = 0.56), diabetes mellitus (*P* for interaction = 0.63), or coronary heart disease (*P* for interaction = 0.47) (Figure 3). However, given the modest sample size in certain stratified subgroups, the absence of significant interaction should be interpreted with caution as reflecting insufficient statistical power to detect subgroup heterogeneity rather than definitive evidence of effect uniformity (Figure 3).

Although no significant correlation was observed between SMI and LVEF in univariate Pearson analysis (*r* = 0.055, *P* = 0.530), SMI showed a significant positive association with LVEF in multivariate regression analysis (*β* = 0.130, *P* = 0.035).

This discrepancy suggests that the relationship between skeletal muscle mass and cardiac systolic function may be confounded by age, BMI, and comorbidities, and becomes evident only after adjustment for these covariates.

## 4 Discussion

In this hospital-based study of elderly male inpatients, we systematically examined the associations of skeletal muscle mass and handgrip strength with cardiac structure and function assessed by echocardiography. Our principal finding was that older patients with reduced skeletal muscle mass, as defined by the low SMI group, exhibited significantly smaller left-sided cardiac chamber dimensions—including LAD, LVEDD, and LVESD—and higher crude PASP compared with their peers with preserved muscle mass. After multivariable adjustment, SMI remained independently and positively associated with LVEDD, LVESD, and LVEF, while the relationship with PASP was attenuated to nonsignificance. This pattern suggests that the elevation in pulmonary pressure observed in the low SMI group is likely attributable to a higher burden of comorbidities and advanced age rather than to a direct, muscle-specific pathway. Importantly, our data also revealed that the association between SMI and LVEF was masked in unadjusted analyses and only became apparent after accounting for age, BMI, and metabolic covariates. This observation underscores the substantial confounding effects of body size and metabolic status on echocardiographic indices of systolic performance and highlights the importance of rigorous covariate adjustment when investigating the muscle-heart relationship in aging populations.

The link between skeletal muscle depletion and alterations in cardiac structure has been increasingly recognized in both population-based and clinical cohorts. In line with our results, Keng and colleagues, using cardiac magnetic resonance imaging in a community-dwelling Asian population, demonstrated that lower appendicular lean mass was significantly associated with reduced left ventricular and left atrial volumes, as well as lower LVEF, independent of age and cardiovascular risk factors (20). Similarly, a large Korean epidemiological study reported that lower skeletal muscle mass was independently associated with smaller LVEDD and LVESD, reinforcing the view that skeletal muscle wasting and cardiac involution progress in parallel as part of a systemic aging phenotype (21). These convergent findings across different ethnic groups and imaging modalities strongly suggest that the association between muscle mass and cardiac chamber size is not merely a reflection of overall body size but rather a specific biological coupling between two striated muscle compartments that share common developmental, metabolic, and endocrine regulatory networks.

Nevertheless, our results appear to diverge from those reported by Wang et al. in a cohort of elderly Chinese patients with established coronary heart disease (CHD), in whom sarcopenia was associated with larger LVEDD and LAD, rather than smaller dimensions (13). This apparent contradiction is instructive and likely stems from key differences in study populations and underlying pathophysiology. In the Wang cohort, all participants had angiographically confirmed obstructive CHD and preserved systolic function (mean LVEF ∼60%), a clinical setting in which sarcopenia may coexist with a state of chronic ischemic burden and neurohormonal activation that favors eccentric left ventricular remodeling and chamber enlargement. In contrast, our study population was considerably older (mean age >82 years), had a lower overall LVEF, and included only a minority of patients with documented CHD (∼32%). In this predominantly very old, non-ischemic cohort, the dominant cardiac phenotype appears to be that of senile involution—characterized by myocyte loss, interstitial fibrosis, and chamber size reduction—rather than ischemia-driven remodeling. This distinction is critical, as it suggests that the direction and magnitude of the muscle–cardiac structure relationship may depend heavily on the underlying cardiac substrate and the relative contributions of aging vs. disease-specific remodeling processes.

One of the most noteworthy findings in our study is the discrepancy between the univariate and fully adjusted models with respect to LVEF. In the crude Pearson analysis, SMI showed no significant correlation with LVEF (*r* = 0.055, *P* = 0.530), yet after adjustment for age, BMI, CHD, diabetes, and hypertension, the association became positive and statistically significant (*β* = 0.130, *P* = 0.035). This inversion—from a null to a positive association—warrants careful interpretation. We propose that this pattern reflects a strong positive confounding effect of BMI and a negative confounding effect of age. SMI is inherently correlated with BMI, which itself is positively associated with cardiac chamber dimensions, but this relationship is not causal with respect to myocardial contractility. Conversely, older age is a powerful determinant of both lower SMI and reduced LVEF through distinct pathways, including sarcopenia progression and myocardial senescence. When these confounders are not controlled for, the true protective or permissive effect of muscle mass on systolic function may be obscured. Only after isolating the independent contribution of SMI by adjusting for these covariates does the intrinsic association emerge. This observation has important methodological implications for future studies: failure to account for body composition and metabolic confounders may lead to underestimation of the muscle–heart axis, particularly in frail, elderly populations where these variables are closely intertwined.

Cardiovascular disease is historically recognized as the primary driver of cardiac structural remodeling. In standard pathological cascades, early-stage stressors typically induce compensatory left ventricular hypertrophy, whereas progression toward decompensation is characterized by eccentric ventricular enlargement and a subsequent decline in ejection fraction. Concurrently, the intrinsic aging process itself dictates cardiac senescence. Regulated by molecular mechanisms such as cardiomyocyte apoptosis, chronic low-grade inflammation, insulin resistance, and neurohormonal dysregulation, senescent hearts undergo a substantial reduction in total cardiomyocyte numbers, compensatory enlargement of remaining cells, and progressive interstitial collagen deposition or fibrosis (22, 23). Given that our study population featured advanced age, overall lower LVEF, and a restricted prevalence of coronary artery disease (only approximately one-third), it is highly plausible that age-induced cardiomyocyte loss and fibrotic involution, rather than classic ischemic remodeling, predominantly governed the structural changes observed herein.

From a pathophysiological perspective, skeletal and cardiac muscles share tightly interconnected biological pathways within the “muscle–heart axis”. Skeletal muscle acts as an endocrine organ, secreting myokines such as irisin (derived from FNDC5) and myostatin, which regulate extracellular matrix remodeling, cardiomyocyte survival, and mitochondrial energy metabolism (24). Age-related skeletal muscle loss disrupts this homeostatic signaling, accelerating low-grade systemic inflammation (“inflammaging”) and oxidative stress, which collectively drive myocardial apoptosis and interstitial fibrosis. Furthermore, specialized molecular regulators, such as the Smyd protein family, serve as essential mediators of skeletal muscle cell differentiation while simultaneously modulating myocardial myofibrillar assembly (25). This shared molecular machinery provides a plausible mechanistic link at the transcriptional and post-translational level, reinforcing the concept of a coordinated decline in both muscle compartments during aging. Notably, while LVEF serves as a conventional measure of global systolic performance, its sensitivity to subtle or early age-related myocardial changes is limited. The attenuation of LVEF associations in unadjusted models and their emergence after adjusting for age and BMI underscore that body size/load and biological age explain a substantial proportion of overall contractility variance, whereas structural skeletal muscle mass maintains a primary, independent link with chamber dimensions (such as LVEDD). Crucially, our data demonstrated that imaging-verified SMI was more independently and reliably associated with left ventricular dimensions and function than dynamic physical performance (handgrip strength). This finding aligns with emerging evidence highlighting distinct physiological roles and diagnostic utilities of muscle mass vs. muscle strength in echocardiographic and ultrasound assessments (26, 27). A temporal mismatch or a relative lag in functional decline may occur during muscle involution, where older individuals in early structural muscle wasting phases preserve motor unit recruitment, thereby maintaining handgrip strength despite lean tissue loss. Consequently, objective mass quantification via DXA serves as a more direct and synchronized surrogate for tracking secondary cardiac structural involution.

Crucially, our data demonstrated that imaging-verified SMI was more independently and reliably associated with left ventricular dimensions and function than dynamic physical performance (handgrip strength). This finding may be explained by a temporal mismatch or a relative lag in functional decline during muscle involution. Specifically, certain elderly inpatients undergoing early or subclinical phases of structural muscle wasting may still preserve individual motor neuron recruitment capabilities, thereby maintaining relatively stable handgrip strength despite an absolute loss of lean tissue mass. This indicates that objective mass quantification via DXA might serve as a more sensitive and synchronized baseline surrogate for tracking secondary cardiac structural involution in this demographic.

From a translational perspective, these findings underscore the clinical relevance of recognizing “cardiosarcopenia” in geriatric management. Early screening using DXA-derived SMI provides crucial risk-stratification information beyond conventional functional testing. Translating these observational insights into clinical practice warrants multimodal therapeutic strategies aimed at skeletal muscle preservation. These include progressive resistance exercise training to induce muscle hypertrophy, targeted nutritional optimization—such as adequate high-quality protein intake (1.2–1.5 g/kg/day) and vitamin D supplementation—and integrated, multidisciplinary geriatric rehabilitation programs to mitigate concomitant cardiac and skeletal muscle decline in aging inpatients.

### 4.2 Limitations

Nevertheless, several limitations should be considered when interpreting the present findings. First, given the cross-sectional design of this study, no causal inference or directional conclusions can be established regarding the relationship between skeletal muscle mass and cardiac structural parameters. Although skeletal muscle depletion and cardiac remodeling may progress concurrently as part of systemic aging, reverse causality remains possible, whereby subtle cardiac dysfunction may contribute to reduced physical capacity and subsequent muscle loss. Prospective longitudinal studies with repeated assessments of muscle mass and cardiac imaging are required to clarify the temporal relationship between these changes. Second, our study population consisted exclusively of older male inpatients, which limits the generalizability of the findings to female populations. Sex-related differences in body composition, hormonal profiles, and cardiovascular aging trajectories may influence the relationship between skeletal muscle status and cardiac remodeling. Therefore, validation in independent cohorts including older women is warranted. Third, several potentially important lifestyle and nutritional factors were not systematically assessed. Specifically, objective measurements of physical activity, exercise habits, dietary intake, and standardized nutritional screening using validated tools such as the Mini Nutritional Assessment Short-Form (MNA-SF) or Malnutrition Universal Screening Tool (MUST) were unavailable. As these factors may influence both skeletal muscle maintenance and cardiovascular structure, residual confounding cannot be completely excluded. Fourth, although major cardiovascular comorbidities were incorporated into the regression models, other unmeasured factors, including frailty status, polypharmacy, and socioeconomic conditions, may have influenced the observed associations. Future studies incorporating comprehensive geriatric assessments and additional clinical variables are needed to further reduce potential confounding. Fifth, echocardiographic parameters, although widely used in clinical practice, are subject to operator dependency and loading conditions, which may introduce measurement variability. Future investigations incorporating cardiac magnetic resonance imaging may provide more precise and reproducible assessments of cardiac chamber geometry and myocardial tissue characteristics. Finally, despite bootstrap validation supporting the stability of the regression models, the relatively modest sample size may have limited statistical power, particularly for subgroup analyses and detection of potential effect modification. Larger multicenter studies are needed to confirm these findings and explore their clinical implications.

## 5 Conclusion

In summary, this study demonstrates that in elderly male inpatients, skeletal muscle mass—assessed by DXA-derived SMI—exhibits a robust, independent, and linear association with left ventricular chamber dimensions and systolic function, even after rigorous adjustment for cardiometabolic comorbidities. Notably, the relationship between muscle mass and cardiac structure appears to be more consistent and diagnostically independent than that of handgrip strength, suggesting that objective muscle mass measurement may offer advantages over functional performance tests when assessing the muscle–heart axis in geriatric populations. These findings lend observational support to the emerging concept of “cardiosarcopenia” as an integrated cardiovascular aging phenotype. However, whether skeletal muscle preservation directly impacts myocardial structural integrity remains to be established. Larger prospective cohort studies and prospective clinical trials are required to validate these cross-sectional associations and clarify the potential therapeutic value of muscle preservation in aging populations.

## Data Availability

The raw data supporting the conclusions of this article will be made available by the authors, without undue reservation.
